# *Triglochin maritima* Extracts Exert Anti-Melanogenic Properties via the CREB/MAPK Pathway in B16F10 Cells

**DOI:** 10.3390/md22120532

**Published:** 2024-11-27

**Authors:** Won-Hwi Lee, Yuna Ha, Jeong-In Park, Won Bae Joh, Mira Park, Jang Kyun Kim, Hee-Kyung Jeon, Youn-Jung Kim

**Affiliations:** 1 Research Institute of Basic Sciences, Incheon National University, Incheon 22012, Republic of Korea; dnjsgnl_@naver.com (W.-H.L.); dbsk335@hanmail.net (Y.H.); qkrwjdls0812@naver.com (J.-I.P.); sinafyou@naver.com (W.B.J.); mira0295@inu.ac.kr (M.P.); jang.kim@inu.ac.kr (J.K.K.); 2 Department of Marine Sciences, Incheon National University, Incheon 22012, Republic of Korea; 3 Advanced Energy Materials and Components R&D Group, Korea Institute of Industrial Technology, Busan 46938, Republic of Korea

**Keywords:** *Triglochin maritima*, halophyte, anti-melanogenesis, tyrosinase, CREB/MAPK pathway, luteolin

## Abstract

*Triglochin maritima*, a salt-tolerant plant, has demonstrated antioxidant effects, the ability to prevent prostate enlargement, antifungal properties, and skin moisturizing benefits. This study aimed to explore the anti-melanogenic potential of the 70% ethanol extract of *T. maritima* (TME) along with its ethyl acetate (TME-EA) and water (TME-A) fractions. TME (10–200 µg/mL), TME-EA (1–15 µg/mL), and TME-A (100–1000 µg/mL) were prepared and applied to B16F10 cells with or without α-MSH for 72 h. MTT assays were used to assess cytotoxicity, and anti-melanogenesis activity was determined by measuring melanin content, conducting a tyrosinase activity assay, and evaluating the expression of melanogenesis-related genes and proteins via RT-PCR and Western blotting. HPLC-PDA was used to analyze TME and TME-EA. The IC_20_ cytotoxicity values of TME, TME-A, and TME-EA without α-MSH, were 198.426 μg/mL, 1000 μg/mL, and 18.403 μg/mL, respectively. TME and TME-EA significantly decreased melanin and tyrosinase activity in α-MSH-stimulated B16F10 cells, with TME-EA showing comparable effects to arbutin, while TME-A showed no influence. TME-EA down-regulated melanogenesis genes (*Tyr*, *Trp1*, *Dct*, *Mitf*, *Mc1r*) and reduced CREB, p-38, and JNK phosphorylation while increasing ERK phosphorylation, suggesting the CREB/MAPK pathway’s role in the anti-melanogenic effect. Luteolin was identified as a potential active ingredient. TME-EA may serve as an effective cosmeceutical for hyperpigmentation improvement due to its anti-melanogenic properties.

## 1. Introduction

Melanocytes are skin cells that synthesize melanin pigments in melanosomes and transport melanin to neighboring keratinocytes through their dendrites [1]. While the quantity of melanin pigment determines skin shade, melanocytes also protect human skin from ultraviolet (UV) rays and various environmental stresses [2,3]. However, excessive production of melanin can cause skin pigmentation diseases, including melasma, age spots, freckles, and malignant melanoma [4]. Meanwhile, the incidence of hyperpigmentation has increased owing to environmental pollution associated with rapid industrialization and excessive exposure to UV and pollutants, leading to alterations in cytokine and hormone production [5,6].

Melanin synthesis occurs in melanosomes. More specifically, eumelanin (brownish black) and pheomelanin (reddish yellow) are produced in melanocytes, a process that is mediated by tyrosinase, tyrosinase-related protein 1 (TRP-1), and dopachrome tautomerase (DCT) [7]. The initial step in the synthesis of eumelanin and pheomelanin involves the oxidation of L-tyrosine to L-3,4-dihydroxyphenylalanine (L-DOPA) by tyrosinase and subsequent oxidation of L-DOPA to L-DOPA quinone (DQ) by tyrosinase. In the presence of sulfhydryl compound, L-cysteine, DQ reacts to generate several cysteinyl-DOPA (CD) isomers, leading to pheomelanin production. Meanwhile, eumelanin is produced through the intramolecular cyclization of DQ and its spontaneous transformation to dopachrome (DC). That is, DC is converted into two dihydroxy indole intermediates, namely, 5,6-dihydroxyindole (DHI) and 5,6-dihydroxyindole-2-carboxylic acid (DHICA). DCT converts DC to DHICA, while DC is decarboxylated to produce DHI. Further oxidation of DHICA and DHI by tyrosinase or TYRP1 produces 5,6-indolequinone (IQ) and indole-5,6-quinone carboxylic acid (IQCA), respectively, which are assembled into the eumelanin pigment.

Several signaling pathways reportedly regulate skin pigmentation. In particular, melanocyte-stimulating hormone (α-MSH) is a melanocortin peptide involved in the induction of melanogenesis. Skin keratinocytes exposed to UV release α-MSH, which increases cAMP levels by binding to the melanocortin 1 receptor (Mc1R) on the surface of melanocytes [8]. Subsequently, protein kinase A (PKA) is activated by cAMP. Activated PKA phosphorylates the cAMP response element-binding protein (CREB), which then phosphorylates microphthalmia-associated transcription factor (MITF)—a key transcription factor in melanin synthesis. Finally, MITF activates the transcription of various melanogenesis related genes, including TYR, TRP1, and DCT [9].

ERK1/2 signaling is involved in the negative feedback mechanism of melanogenesis. That is, elevated cAMP activates ERK1/2 signaling and induces ubiquitin-dependent proteasomal degradation of MITF. Accordingly, degradation of MITF via ERK1/2 signaling has been identified as a promising target for developing anti-melanogenic compounds [10,11,12]. Moreover, the phosphorylation of p38 MAPK and c-Jun N-terminal kinases (JNK) increases melanogenesis through up-regulation of MITF and TYR, suggesting that the regulation of the CREB and MAPK signaling pathways in melanocytes to suppress MITF transcription represents an important target for melanogenesis inhibition [13,14]. The main mechanism-based targets involved in suppressing melanin production are being actively investigated to discover phytochemicals with anti-melanin activity, and this study was also conducted with a focus on these studies.

Halophytes are found in many parts of the world and are important for preserving and maintaining the natural balance of ecosystems, particularly in tidal flat ecosystems, where they provide crucial habitats. However, halophytes also contain various active compounds that have potential health benefits owing to their unique growth environments [15,16,17]. For instance, *Triglochin maritima* L. is a perennial halophyte from the Juncaginaceae family [18] that inhabits temperate regions of the Northern Hemisphere, and in South Korea, where it is distributed in clusters along the east and west coasts and on Jeju Island [19]. Reportedly, *T. maritima* also has antioxidant and antifungal properties while proving effective at preventing prostate enlargement and moisturizing skin [20,21,22]. However, studies on the anti-melanogenic effects of *T. maritima* extracts are lacking.

This study was designed to examine the anti-melanogenic effects of *T. maritima* extracts in α-MSH-induced B16F10 cells and explore their bioactive compounds. Hence, the main objective was to characterize the phytochemicals in *T. maritima* with potential applications in cosmetics, functional foods, and pharmaceuticals.

## 2. Results

### 2.1. Cell Viability of B16F10 Cells Exposed to T. maritima Extracts

**Figure 1:**
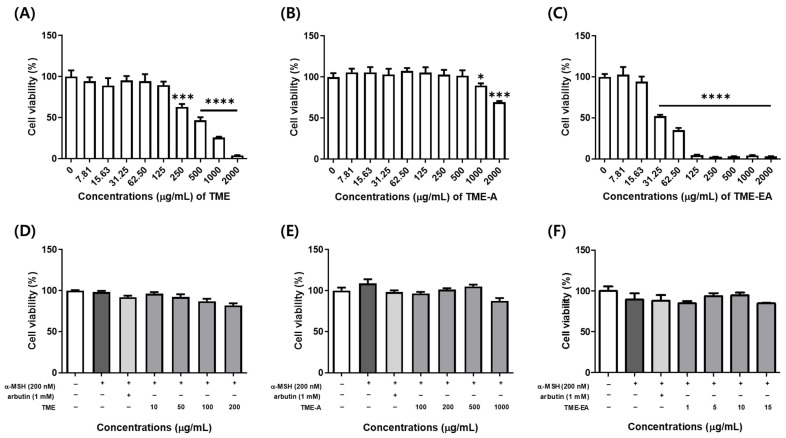
Cytotoxicity of B16F10 cells following treatment with TME, TME-A, and TME-EA. The cells were treated with TME (**A**), TME-A (**B**), and TME-EA (**C**) for 24 h without α-MSH or with 10, 50, 100, and 200 µg/mL of TME (**D**), 100, 250, 500, and 1000 µg/mL of TME-A (**E**), and 1, 5, 10, and 15 µg/mL of TME-EA (**F**) with 200 nM α-MSH for 72 h. Arbutin (1 mM) was used as a positive control. The results are expressed as a percentage of the value derived for the control group. Values are presented as means ± SEM ((**A**–**C**): n = 6, (**D**–**F**): n = 3) and statistical analysis was performed using the Welch ANOVA test with the Games–Howell multiple comparison test ((**A**–**C**)) and the Kruskal–Wallis test with Dunn’s multiple comparison test ((**D**–**F**)). Statistical significance is indicated as follows: * *p* < 0.05, *** *p* < 0.001, and **** *p* < 0.0001 compared to the control group.

To assess the potential toxicity of *T. maritima* extracts, an MTT assay was performed with B16F10 cells subjected to a range of extract concentrations (7.8125–2000 μg/mL) with and without α-MSH. The IC_20_ values for TME, TME-A, and TME-EA without α-MSH, were 198.426 μg/mL, 1000 μg/mL, and 18.403 μg/mL, respectively (Figure 1A–C). Based on these cytotoxicity results, melanin content was compared and tyrosinase activity was evaluated in subsequent experiments by treating TME, TME-A, or TME-EA with α-MSH at the highest concentration in which no cytotoxicity was observed in any experimental group (Figure 1D–F).

### 2.2. Effects of T. maritima Extracts on Melanogenesis in α-MSH-Stimulated Melanoma Cells

**Figure 2:**
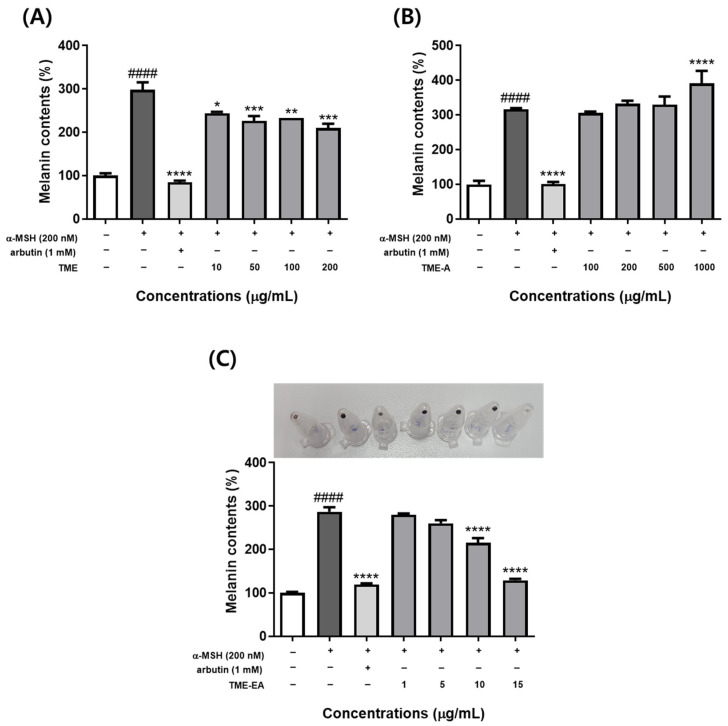
Inhibitory effect of TME, TME-A, and TME-EA on melanin synthesis in B16F10 cells. Cells were treated with α-MSH (200 nM), arbutin (1 mM), TME (**A**), TME-A (**B**), or TME-EA (**C**) for 72 h. Melanin content was measured based on absorbance at 405 nm. Values are expressed as means ± SEM (n = 3) and statistical analysis was performed using one-way ANOVA with Tukey’s multiple comparison test. #### *p <* 0.0001 versus control group, * *p <* 0.05, ** *p <* 0.01, *** *p <* 0.001, and **** *p <* 0.0001 versus α-MSH treatment group.

To assess the influences of TME, TME-A, and TME-EA on melanogenesis, B16F10 melanoma cells were treated with each extract and α-MSH for 72 h, followed by measurement of intracellular melanin levels. The results showed that α-MSH treatment increased melanin content in B16F10 cells (Figure 2). Meanwhile, TME-A (Figure 2B) exhibited no inhibitory activity on melanin synthesis, whereas TME (Figure 2A) and TME-EA (Figure 2C) treatment significantly reduced melanin content compared to B16F10 cells stimulated with only α-MSH (*p <* 0.05). In particular, TME-EA induced a similar inhibitory effect as that elicited by arbutin (positive control). In comparison to the untreated control, the α-MSH-stimulated control had a melanin content of 286.94%, while the positive control and 15 μg/mL TME-EA had values of 119.406% and 127.239%, respectively. Moreover, the amount of pigment in the cell pellet exhibited a proportional decrease relative to changes in melanin content. These results suggest that *T. maritima* extract and the TME-EA fraction regulate melanin production.

### 2.3. Tyrosinase Activity by T. maritima Extract in α-MSH-Induced B16F10 Cells

**Figure 3:**
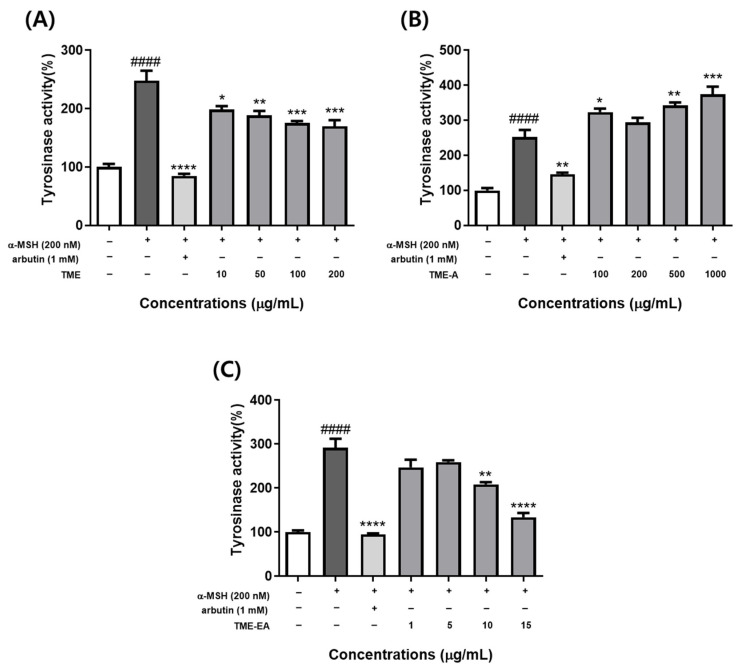
Effect of TME, TME-A, and TME-EA on intracellular tyrosinase activity. Cells (1.0 × 104 cells/mL) were pre-incubated for 24 h and treated with TME (**A**), TME-A (**B**), TME-EA (**C**), or 200 nM α-MSH for 72 h, and intracellular tyrosinase activity was measured. Values are expressed as means ± SEM (n = 3) and statistical analysis was performed using one-way ANOVA with Tukey’s multiple comparison test. #### *p <* 0.0001 versus control group, * *p <* 0.05, ** *p <* 0.01, *** *p <* 0.001, and **** *p <* 0.0001 versus α-MSH treatment group.

Tyrosinase is an important enzyme for melanin synthesis. Hence, the tyrosinase inhibitory effect was measured to determine whether the extracts had a direct effect on tyrosinase activity in B16F10 cells. Results show that α-MSH significantly increased tyrosinase activity (*p <* 0.001) and TME-A (Figure 3B) also notably enhanced tyrosinase activity. By contrast, TME (Figure 3A) and TME-EA (Figure 3C) decreased cell tyrosinase activity in a concentration-dependent manner. TME-EA, in particular, exhibited tyrosinase inhibitory effects similar to those of arbutin. Compared to the control, the tyrosinase activity was increased to 291.318% in the α-MSH-stimulated control and decreased to 94.907% and 133.98% in cells treated with arbutin or 15 μg/mL TME-EA, respectively. These results suggest that TME-EA effectively inhibits tyrosinase activity, thereby suppressing melanin production in B16F10 cells.

### 2.4. Changes in Melanogenesis-Related Gene Expression Induced by TME-EA

**Figure 4:**
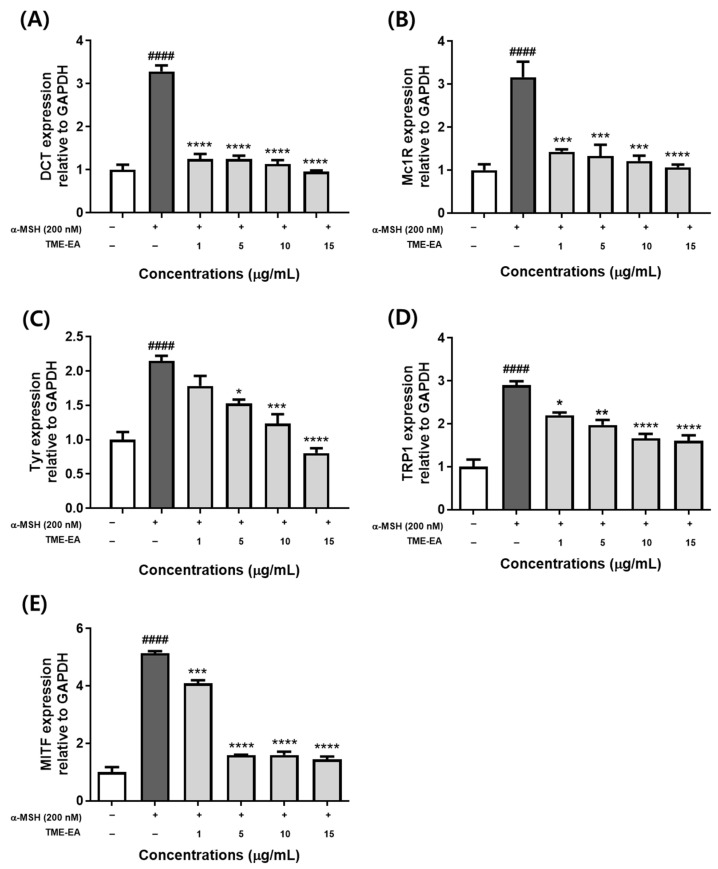
Effect of TME-EA on genes related to melanogenesis. B16F10 cells were treated with TME-EA (1, 5, 10, and 15 µg/mL) and α-MSH (200 nM). mRNA levels of *Tyr* (**A**), *Dct* (**B**), *Trp1* (**C**), *Mc1r* (**D**), and *Mitf* (**E**) were determined using RT-qPCR. GAPDH was used as a reference gene. Data represent means ± SEM (n = 3) and statistical analysis was performed using one-way ANOVA with Tukey’s multiple comparison test. #### *p <* 0.0001 versus control group, * *p <* 0.05, ** *p <* 0.01, *** *p <* 0.001, and **** *p <* 0.0001 versus α-MSH treatment group.

The effect of TME-EA on the transcription of key melanogenesis-associated genes (*Tyr*, *Trp1*, *Dct*, *Mitf*, and *Mc1r*) was evaluated by treating B16F10 cells with various concentrations of TME-EA (1, 5, 10, and 15 µg/mL) and 200 nM α-MSH for 24 h, followed by quantitative real-time PCR analysis of mRNA levels. The expression of *Dct* (Figure 4A) and *Mc1r* (Figure 4B) mRNA exhibited a decreasing tendency at all tested concentrations. The expression of *Tyr* (Figure 4C) and *Trp1* (Figure 4D) was down-regulated in a concentration-dependent manner. In addition, *Mitf* (Figure 4E) expression was up-regulated by α-MSH and significantly inhibited by 5, 10, and 15 µg/mL of TME-EA. These results suggest that the ethyl acetate fraction of *T. maritima* regulates the transcription of melanogenesis-related genes.

### 2.5. Effects of TME-EA on Melanogenesis-Related Signaling Pathways

**Figure 5:**
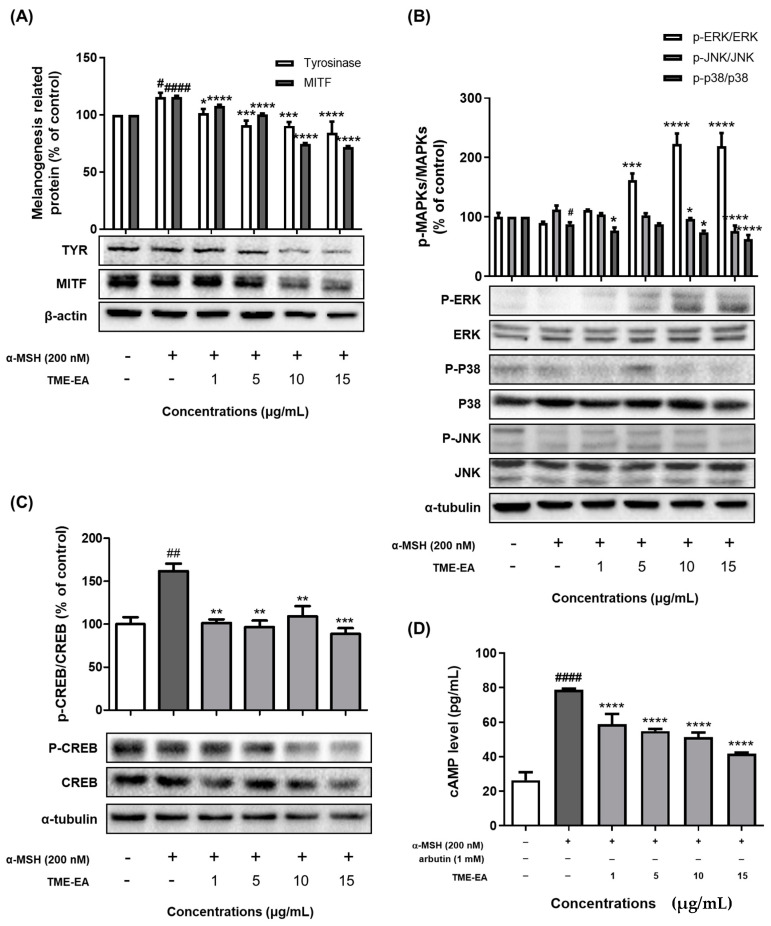
Effect of TME-EA on melanogenesis-related signaling pathways. B16F10 cells were treated with TME-EA (1, 5, 10, and 15 µg/mL) in the presence of α-MSH (200 nM) for 24 h. MITF and tyrosinase levels (**A**), MAPK protein phosphorylation (**B**), CREB phosphorylation (**C**), and cAMP levels (**D**) were assessed. Values are presented as means ± SEM (n = 3) and statistical analysis was performed using one-way ANOVA with Tukey’s multiple comparison test. # *p <* 0.05, ## *p* < 0.01, #### *p <* 0.0001 versus control group, * *p <* 0.05, ** *p <* 0.01, *** *p <* 0.001, and **** *p <* 0.0001 versus α-MSH treatment group.

Cells were treated with TME-EA (1, 5, 10, and 15 µg/mL) in the presence of α-MSH for 24 h, and the phosphorylation status of CREB and MAPK family members (ERK, P38, JNK) was examined in the cell lysates. Tyrosinase and MITF protein levels exhibited a concentration-dependent reduction (Figure 5A). Additionally, ERK phosphorylation was up-regulated, while p38 and JNK phosphorylation showed a decreasing trend (Figure 5B). The phosphorylation of CREB (Figure 5C) was also reduced in a dose-dependent manner. Correspondingly, intracellular cAMP levels (Figure 5D) decreased as TME-EA concentration increased. These findings indicate that TME-EA may regulate melanogenesis by promoting MITF degradation via the CREB/MAPK signaling pathway.

### 2.6. Identification of Active Compounds in T. maritima Extract

**Figure 6:**
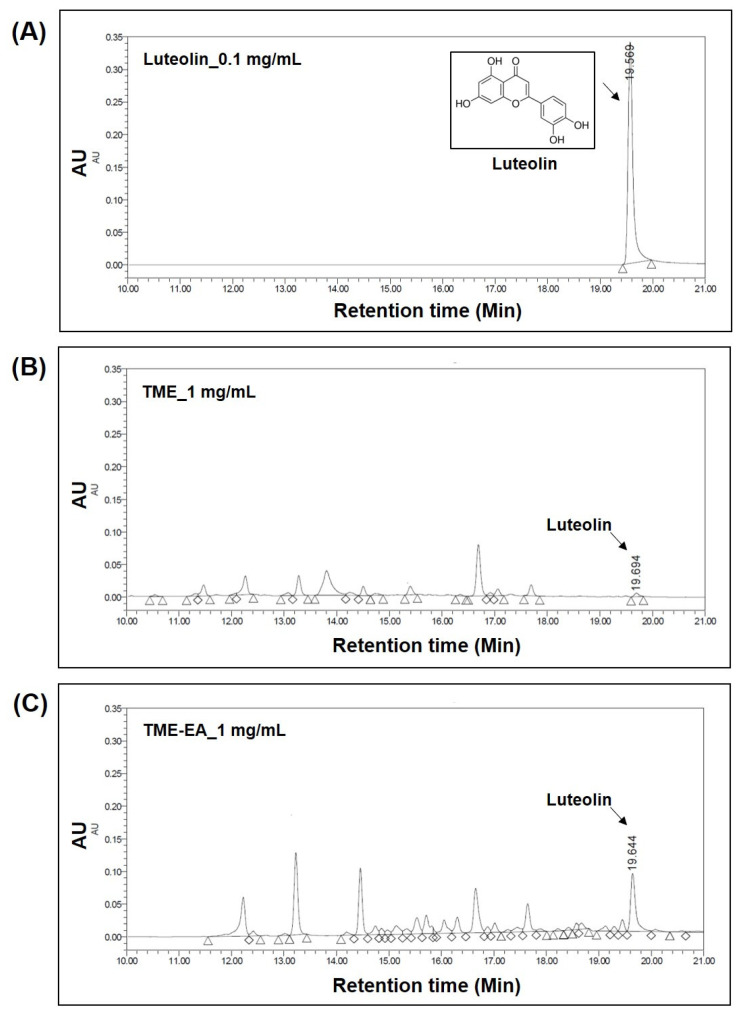
Measurement of active compounds in TME and TME-EA at 254 nm using HPLC-PDA analysis. Luteolin standard (0.1 mg/mL) (**A**); TME (1 mg/mL) (**B**); TME-EA (1 mg/mL) (**C**).

The active ingredients in TME and TME-EA were analyzed. Peak identification was performed by comparing the retention time and UV spectra indicated in the chromatograms with the spectrum of the standard compound. Peaks consistent with standard luteolin were identified in the chromatograms of TME and TME-EA (Figure 6, Figure S1). Quantitative analysis of luteolin in TME and TME-EA was performed using a standard curve. TME (1 mg/mL) contained 1.602 μg/mL luteolin, while TME-EA (1 mg/mL) contained 14.957 μg/mL luteolin. The amount of luteolin in each extract, converted to mg per 1 g of the original plant material (*T. maritima*), was calculated as 0.212 mg/g for TME and 0.121 mg/g for TME-EA. This difference is attributed to the lower yield of TME-EA extract (0.008 g/g, 0.81%) compared to TME extract (0.133 g/g, 13.25%) from 1 g of *T. maritima*. These results imply that the ability of *T. maritima* extract to inhibit melanin production might be due to the presence of luteolin.

### 2.7. Effect of Luteolin on Melanogenesis in α-MSH-Induced B16F10 Cells

**Figure 7:**
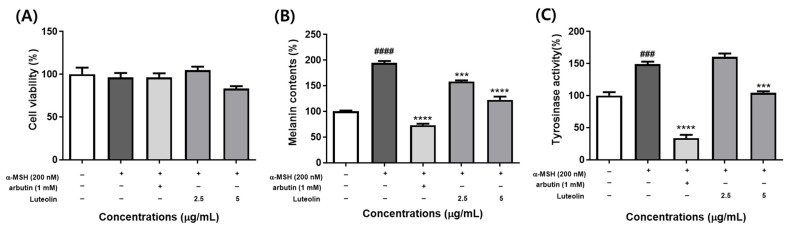
Effect of luteolin on cytoxicity, melanin synthesis, and intracellular tyrosinase activity. Cells were treated with luteolin or 200 nM α-MSH for 72 h, and cell viability (**A**), melanin content (**B**), and intracellular tyrosinase activity (**C**) was assessed. Values are expressed as means ± SEM (n = 3) and statistical analysis was performed using one-way ANOVA with Tukey’s multiple comparison test. ### *p <* 0.001, #### *p <* 0.0001 versus control group and *** *p <* 0.001, **** *p <* 0.0001 versus α-MSH treatment group.

We then sought to determine whether luteolin, contained in TME and TME-EA, affected anti-melanogenesis via a mechanism similar to that of TME and TME-EA. When treated with the highest concentration of luteolin, the cytotoxicity in B16F10 cells showed 83.14% ± 4.985% compared to the control group and one-way ANOVA followed by Dunnett’s multiple comparison tests revealed no significant differences. The melanin content in the α-MSH-induced control, arbutin-, and 5 μg/mL luteolin-treated groups was 194.397%, 73.275%, and 122.414%, respectively, relative to the untreated control. Furthermore, tyrosinase activity levels were 148.936%, 35.638%, and 104.448% for the α-MSH-induced control, arbutin-, and luteolin-treated groups, respectively, compared to the control. These results suggest that inhibition of tyrosinase activity by luteolin reduces melanin production in B16F10 cells (Figure 7).

## 3. Discussion

In this study, *T. maritima* was extracted and fractionated to investigate its potential as an additive for skin whitening in functional cosmetics and pharmaceuticals. Several studies have demonstrated that *T. maritima* extract exhibits antioxidative, antibacterial, and prostatic hyperplasia prevention effects. However, studies on the active ingredients of *T. maritima* have not yet been carried out.

Given that cytotoxicity is a concern for all potential additives, before examining the effect on melanin production in B16F10 melanoma cells, cytotoxicity was measured using the MTT assay. The IC_20_ values of the TME, TME-A, and TME-EA fractions were 198.426, 1000, and 18.403 µg/mL, respectively. Hence, subsequent analyses were performed using concentrations within the IC_20_ for each fraction (TME: 10, 50, 100, and 200 µg/mL; TME-A: 100, 250, 500, and 1000 µg/mL; TME-EA: 1, 5, 10, and 15 µg/mL). Some of the current skin whitening products have high cytotoxicity, lack penetrability, and can cause skin sensitivity and contact dermatitis. Hence, due to safety concerns about the use of melanin inhibitors, there has been an increased focus on discovering natural substances with similar properties [23].

Tyrosinase is a rate-limiting enzyme that regulates melanogenesis, catalyzing the initial steps of melanin biosynthesis [24]. As such, increased melanogenesis is directly linked to enhanced tyrosinase activity. To determine whether *T. maritima* extracts suppress melanin production by inhibiting tyrosinase activity, we conducted experiments using α-MSH-induced melanoma cells. Our results confirmed that TME and TME-EA inhibited tyrosinase activity in a concentration-dependent manner. Notably, the inhibitory effect of TME-EA at 15 μg/mL was comparable to that of the positive control, arbutin. These results suggest that *T. maritima* extract may effectively inhibit α-MSH-induced melanogenesis through down-regulating tyrosinase activity, thereby reducing cellular melanin synthesis.

When α-MSH binds MC1R, adenylate cyclase becomes activated, increasing the intracellular cAMP levels. [25]. As cAMP levels in melanocytes increase, the CREB signaling pathway is activated, which induces the expression of proteins involved in melanogenesis. The authors of [26] reported that activation of the CREB signaling pathway up-regulates the expression of MITF and induces the expression of TRP-1, TRP-2, and tyrosinase. Indeed, MITF is a tyrosinase-regulated transcription factor that plays a crucial role in the regulation of melanin synthesis, and MITF activity is regulated through the MAPK signaling pathway, including ERK, known as negative feedback [27]. Therefore, inhibition of this process is an important strategy for inhibiting melanin synthesis in melanocytes. To investigate the effect of *T. maritima* extracts on these pathways, we determined the expression of *Mc1r*, *Tyr*, *Trp1*, *Trp2*, and *Mitf* mRNA, i.e., the major enzymes involved in melanin synthesis. Treatment with TME-EA significantly down-regulated the expression of *Tyr*, *Trp1*, *Mc1r*, and *Dct* mRNA at all concentrations, while that of *Mitf* was decreased following treatment with 5, 10, or 15 μg/mL TME-EA. Subsequent Western blotting analysis revealed that the abundance of tyrosinase and MITF were lower following treatment with TME-EA compared with those in the α-MSH-treated group at all concentrations.

Additionally, to ascertain the mechanism underlying the activity of *T. maritima* extracts in α-MSH-induced B16F10 cells, the abundance of CREB, ERK, p38, and JNK was assessed. A tendency to decrease phosphorylated CREB was observed at all concentrations of TME-EA compared to the α-MSH treatment group. Moreover, the abundance of cAMP—a key factor upstream of CREB—also decreased. Further analysis of the effect of TME-EA on MAPK in α-MSH-induced B16F10 cells revealed that phosphorylated ERK tended to increase in a concentration-dependent manner. An increase in ERK mediates MITF degradation [10,11]. In addition, phosphorylated p38 and JNK levels were significantly suppressed following treatment with 15 μg/mL TME-EA compared with the α-MSH-treated group.

Similar to our findings, Seong et al. [28] reported that *Cryptotaenia japonica* suppresses melanogenesis via CREB- and MAPK-related signaling pathways in B16F10 cells. Moreover, Alam et al. [29] found that *Nymphaea nouchali* flower extract decreases melanogenesis via regulation of cAMP/MAPKs/CREB signaling and proteasomal degradation of MITF in B16F10 cells. Regarding halophytes, *Arthrophytum scoparium* reportedly inhibits melanogenesis by down-regulating tyrosinase and melanogenesis-related gene expression through the cAMP/PKA pathway [30]. Additionally, *Limonium tetragonum* extract suppresses melanogenesis via down-regulating the expression of tyrosinase, TRP-1, MITF, and DCT [31]. Collectively, these findings suggest that *T. maritima* inhibits melanogenesis by blocking the activation of CREB/p38/JNK and promoting that of ERK in α-MSH-treated B16F10 cells.

Halophytes live in salty environments, such as brackish waters and tidal flats, and promote the synthesis and accumulation of polyphenols as they grow under high-salinity conditions [32]. Halophytes also contain many bioactive substances, including polyphenols, flavonoids, sterols, and amino acids [33]. In particular, *Limonium effusum* and *Limonium sinuatum* reportedly have antibacterial and antioxidant activities [34], while *Suaeda fruticosa* has demonstrated antioxidant, anti-inflammatory, and anticancer effects [35]. Hence, halophytes represent potential sources for the cosmetic and pharmaceutical industries.

HPLC analysis identified various peaks in the *T. maritima* extract, among which luteolin, a flavonoid, was identified. Luteolin, a flavonoid that can be used in food, cosmetics, and pharmaceuticals, has antioxidant [36], anti-inflammatory [37], anti-allergic [38], anticancer [39], and anti-melanogenesis effects. In particular, luteolin is known to decrease cAMP levels, thereby inhibiting melanin content and tyrosinase activity [40,41]. Calvo et al. (2023) identified luteolin as a major flavonoid in the ethanol extract of *T. maritima*, with the amounts ranging from 34.66% to 8.48% in specific extract fractions. The study also reported that luteolin from *T. maritima* exhibits strong PEP-inhibitory activity, attributed to structural features such as hydroxyl groups located at specific positions on the B-ring and A-ring. Furthermore, luteolin, alongside other flavonoids such as apigenin and chrysoeriol, was shown to enhance the antioxidant and enzyme inhibitory activities of the extract. In rat models, luteolin demonstrated antihypertensive properties, potentially through mechanisms beyond ACE inhibition. Consistent with these findings, this study highlights the critical role of luteolin in the bioactivity of *T. maritima* extracts and underscores its potential applications in cosmetics and nutraceuticals. To achieve this, further investigation into the biological activity of luteolin isolated from *T. maritima* extracts is needed. Additionally, the anti-melanogenic activity of luteolin isolated from *T. maritima* extract should be evaluated using in vivo models.

This study has several limitations. First, we did not identify or quantify other potential active ingredients except luteolin, because comprehensive ingredient analysis (e.g., LC-MS/MS), including various fractionation of the TME, was not conducted. The TME-EA fraction exhibited the strongest activity, and the higher concentration of luteolin in this fraction indirectly supports its role as a bioactive compound. Future research should include fractionation and detailed analyses to uncover additional anti-melanogenic agents. Notably, Calvo et al. [42] employed HPLC-ESI-QTOF-MS to analyze active fractions in ethanol extracts of *T. maritima*, demonstrating PEP inhibition, antioxidant activity, and antihypertensive effects, and identified luteolin as a major constituent. These findings complement our results and enhance the reliability of luteolin’s biological role in the extracts. Second, further research is needed to analyze the composition and active compound content of *T. maritima* extracts collected from different seasons and locations. This is essential, as numerous studies have reported variability in the bioactive compound profiles due to environmental factors. Finally, in order to utilize and confirm luteolin or *T. maritima* extracts as whitening ingredients in cosmetic products, it is necessary to perform in vivo experiments using zebrafish and skin clinical studies.

## 4. Materials and Methods

### 4.1. Preparation of T. maritima Extracts

*Triglochin maritima* L. was collected from Dongmak beach in Incheon, South Korea, thoroughly rinsed twice with tap water to eliminate salt and soil, and then carefully washed with distilled water, immediately dried, and maintained at −20 °C until use. Thereafter, the frozen samples were homogenized with a grinder to obtain *T. maritima* powder. *T. maritima* samples were then treated with 70% ethanol (3.1 L), which was 10 times the sample weight (309.4 g), and subjected to immersion extraction at 24 °C for 48 h. Subsequently, the extracts were filtered using Whatman filter paper no. 2 (Whatman, Maidstone, UK) and concentrated using a rotary vacuum concentrator (Sunileyela, Sungnam, South Korea). Following lyophilization, 35.7 g of 70% ethanol extract (TME) was obtained, which underwent fractionation using an EtOAc–water solution (1:1 *v*/*v*) to produce the EtOAc (TME-EA) and water (TME-A) fractions. The fractionated solutions were concentrated using a vacuum concentrator. After lyophilization, 2.19 g of an EtOAc fraction and 28.23 g of a water fraction were obtained. Before conducting the experiments, *T. maritima* extracts and fractions were dissolved in dimethyl sulfoxide (DMSO). The group treated with only DMSO without the extract was defined as the control group.

### 4.2. Cell Culture

The murine melanoma cell line B16F10 was purchased from the American Type Culture Collection (ATCC, Rockville, MD, USA; CRL-6475). Cells were cultured in Dulbecco’s modified Eagle’s medium (DMEM; Gibco, Grand Island, NY, USA; Cat. # 12800-017) with 10% heat-inactivated fetal bovine serum (FBS; Grand Island, NY, USA; Cat. # 16000-044) and 1% penicillin–streptomycin (Gibco, Grand Island, NY, USA; Cat. # 15140-122) at 37 °C in a 5% CO_2_ incubator. The cell medium was refreshed every two days. B16F10 cells were starved for 24 h.

### 4.3. Cell Viability

Cell viability was evaluated according to the method described by Seo et al. [13]. To determine the effect of *T. maritima* extracts on cell viability, the 3-(4,5-dimethylthiazol-2-yl)-2,5-diphenyltetrazolium bromide (MTT; Sigma-Aldrich, St. Louis, MO, USA; Cat. # M2128) assay was used. Briefly, B16F10 cells were seeded at a density of 5 × 10^4^ cells/mL in a 96-well plate for 24 h and treated with different concentrations of *T. maritima* extracts for 24 h. For the melanin content assay, B16F10 cells were cultured in a 24-well plate at a density of 1 × 10^4^ cells/mL for 24 h and then incubated with a medium containing various *T. maritima* extracts or 1 mM arbutin—a known melanin inhibitor (Sigma-Aldrich, St. Louis, MO, USA; Cat. #A4526)—with or without 200 nM α-MSH (Sigma-Aldrich, St. Louis, MO, USA; Cat. #M4135) for 72 h. After washing with phosphate-buffered saline (PBS; Gibco, Grand Island, NY, USA), the cells were incubated with MTT solution (0.5 mg/mL) for 3 h. Subsequently, the formazan crystals produced by the MTT solution were dissolved in 1 mL of DMSO, and the absorbance was measured at 570 nm using a Varioskan LUX Multimode microplate reader (Thermo Fisher Scientific, Waltham, MA, USA). The results were compared with those of the control group to determine the cell viability.

### 4.4. Measurement of Melanin Content

The measurement of melanin content was performed following the method described by Seo et al. [13]. B16F10 cells were cultured in a 24-well plate at a density of 1 × 10⁴ cells/mL and incubated for 24 h. The cells were then treated with various concentrations of *T. maritima* extracts (10–200 µg/mL of TME, 100–1000 µg/mL of TME-A, and 1–15 µg/mL of TME-EA) or 1 mM arbutin for 72 h, with or without 200 nM α-MSH. After washing with PBS, the cells were lysed using 1% Triton X-100 (Sigma-Aldrich, St. Louis, MO, USA) containing 1 mM phenylmethylsulfonyl fluoride (PMSF; Bio-Rad Hercules, CA, USA). The lysates were processed by freezing and thawing three times and centrifuged at 13,000 rpm for 15 min at 4 °C. The pellets were dissolved in a 1 N NaOH and 10% DMSO solution in distilled water at 80 °C for 1 h. Absorbance was measured at 405 nm using a microplate reader, and the melanin content was quantified using a standard curve generated for synthetic melanin (Sigma-Aldrich, St. Louis, MO, USA; Cat. # M0413). To calculate the melanin content per μg of protein obtained from each cell, we determined the protein concentration using the Pierce™ BCA protein assay kit (Thermo Fisher Scientific, Waltham, MA, USA; Cat. # 23225) with bovine serum albumin (BSA) as the standard.

### 4.5. Cellular Tyrosinase Activity Assay

The supernatant from cell lysates was obtained in the same way as described above and used as the crude tyrosinase extract for the activity assay. Protein concentration was determined using a BCA protein assay kit. In a 96-well plate, a reaction mixture was prepared comprising 40 µg of protein (adjusted to 100 µL with supernatant) and 100 µL of 3 mM L-DOPA (from Sigma-Aldrich, St. Louis, MO, USA; Cat. # D9628) per well. The mixture was incubated at 37 °C for 1 h, and the absorbance was measured at 475 nm using a microplate reader. Tyrosinase activity was quantified using Equation (1):

(1) $$\mathrm{Tyrosinase}\;\mathrm{activity}\;(\%)=\left(\frac{475\;\mathrm{nm}\;\mathrm{Absorbance}\;\mathrm{of}\;\mathrm{with}\;\mathrm{extract}\;\mathrm{treatment}}{475\;\mathrm{nm}\;\mathrm{Absorbance}\;\mathrm{of}\;\mathrm{without}\;\mathrm{extract}\;\mathrm{treatment}}\right)\;\times \;100$$

### 4.6. RNA Isolation and cDNA Synthesis

B16F10 cells were cultured in 100 mm dishes at a concentration of 10 × 10^4^ cells/mL and treated with *T. maritima* extracts in the presence or absence of 200 nM α-MSH for 24 h. Total RNA was extracted from the sample using TRIzol™ reagent (Invitrogen, Carlsbad, CA, USA), and RNA concentration and purity were evaluated at 260/280 nm using a NanoDrop^®^ spectrophotometer (Daemyeong Science, Seoul, South Korea). The RNA was further purified with an RNeasy Mini Kit (Qiagen, Hilden, Germany), and complementary DNA was obtained by reverse-transcribing the purified RNA using the ReverTra Ace™ qPCR RT master mix with gDNA remover (Toyobo, Osaka, Japan) according to the manufacturer’s instructions.

### 4.7. Real-Time qPCR Analysis

The expression levels of target genes were quantified via real-time quantitative PCR (RT-qPCR) using Thunderbird™ SYBR^®^ qPCR Mix (Toyobo, Osaka, Japan) and a CFX96™ Real-Time System (Bio-Rad, Hercules, CA, USA). The thermocycling protocol comprised 45 cycles of 95 °C for 3 min, 95 °C for 1 min (denaturing), 59–63.3 °C for 30 s (annealing), and 72 °C for 30 s (primer extensions). The primer sequences were obtained from Bioneer (Daejeon, South Korea) and are shown in Table 1. The ΔΔCq method was applied to calculate the relative expression of the target genes with Gapdh as the internal control for normalization.

### 4.8. Western Blotting

B16F10 cells were cultured in 100 mm dishes at a density of 10 × 10^4^ cells/mL and exposed to *T. maritima extracts* for 24 h, with or without 200 nM α-MSH. The cells were then lysed using RIPA buffer containing a phosphatase inhibitor (Sigma-Aldrich, St. Louis, MO, USA) and 1 mM PMSF. Protein concentrations were calculated using a BCA protein assay kit. The lysates (30 μg) were separated by SDS-PAGE and transferred to a polyvinylidene fluoride (PVDF; Bio-Rad, Hercules, CA, USA) membrane, which was blocked using 5% skim milk (Bio-Rad, Hercules, CA, USA) or 2%–5% BSA (Sigma-Aldrich, St. Louis, MO, USA) in Tris-T buffer containing 0.1% Tween 20. Subsequently, the membranes were incubated at 4 °C overnight with primary antibodies against CREB (1:1000, rabbit mAb), p-CREB (1:1000, rabbit mAb), ERK (1:1000, rabbit mAb), p-ERK (1:1000, rabbit mAb), p38 (1:1000, rabbit mAb), p-p38 (1:1000, rabbit mAb), JNK (1:1000, rabbit mAb), p-JNK (1:1000, rabbit mAb), and tyrosinase (1:1000, mouse mAb), MITF (1:1000, rabbit mAb), tubulin (1: 2000, rabbit mAb), and β-actin (1: 2000, rabbit mAb). All primary antibodies were purchased from Cell Signaling Technology (Danvers, MA, USA). They were then incubated with anti-rabbit horseradish peroxidase (HRP) (1:2000; Cell Signaling Technology, Danvers, MA, USA) or anti-mouse horseradish peroxidase (HRP) (1:2000; Sigma-Aldrich, St. Louis, MO, USA) secondary antibodies at 4 °C for 1 h. Protein abundance was visualized using Clarity Western ECL Substrate (Bio-Rad, Hercules, CA, USA). Band strength was measured with the ChemiDoc™ XRS+ System (Bio-Rad, Hercules, CA, USA). The loading quantity was normalized using α-tubulin and β-actin, and all experiments were repeated three times.

### 4.9. Enzyme-Linked Immunosorbent Assay (ELISA)

To measure cAMP levels, a cAMP Parameter Assay Kit (R&D Systems, Minneapolis, MN, USA) was used. B16F10 cells were treated with *T. maritima* extracts, collected, and rinsed three times with cold PBS. The resulting cell lysates were added to microplates coated with goat–mouse antibody. Subsequently, the monoclonal antibody was added and incubated for 1 h at 24 °C on a shaker set to 500 ± 50 rpm. The wells were washed four times with wash buffer to remove any remaining monoclonal antibodies. Standard and cAMP conjugate were then added to each well and incubated for 2 h at 24 °C on a shaker. After washing, a substrate was added to measure the bound enzyme activity, and the color change was terminated before the absorbance was measured at 450 nm using a microplate reader.

### 4.10. High-Performance Liquid Chromatography (HPLC) Analysis

*T. maritima* extracts were analyzed using HPLC to identify the active components. A Waters Alliance 2690/2695 HPLC with a Waters 2996 PDA Detector (Waters Corp., Milford, MA, USA) and Empower 2 Software Build 2154 (Waters Corp., Milford, MA, USA) were used to acquire the chromatographic data. A Capcell Pak UG120 C18 column (150 mm × 4.6 mm, 3 μm, Shiseido, Tokyo, Japan) was used for chromatographic separation at a flow rate of 0.2 mL/min and column temperature of 40 °C. The injection volume was 10 μL, and the UV wavelength was set to 349 nm. The mobile phase comprising A (0.1% formic acid in water) and B (acetonitrile) was filtered using a 0.45 μm filter. The gradient profile is presented in Table 2. The peaks in the chromatogram were recognized by comparing the retention times and UV spectra with those of reference materials. The TME and TME-EA samples were utilized at 10 mg/mL, and luteolin (Sigma-Aldrich, St. Louis, MO, USA) was used as the standard at a concentration of 1 mg/mL.

### 4.11. Statistical Analysis

Data analysis was performed using the Statistical Analysis System software (PRISM, GraphPad Software 10, San Diego, CA, USA). The results are presented as means ± standard error of the mean (SEM). Comparisons between groups were performed using one-way analysis of variance (ANOVA) followed by Tukey’s multiple comparison post hoc test. *p <* 0.05 was considered significant. Experimental results for cell viability, melanin content, tyrosinase activity, cAMP levels, real time qPCR, and Western blot analysis (n = 3, comparing α-MSH- and extract-treated groups) satisfied normality on the Shapiro–Wilk test and homogeneity of variance on the Brown–Forsythe test. In these cases, one-way ANOVA followed by Tukey’s post hoc test was performed to evaluate differences between groups. When the assumption of normality was satisfied, but homogeneity of variance was not, Welch’s ANOVA followed by the Games–Howell test was conducted. Additionally, for data that did not meet the assumptions of normality or homogeneity of variance, the Kruskal–Wallis test followed by Dunn’s post hoc test was applied. The statistical analysis methods applied to each result are detailed in the figure legends.

## 5. Conclusions

The results of this study provide the first evidence of the anti-melanogenic effect of *T. maritima*, establishing the CREB/MAPK pathway as a key mediator of this activity, as shown in the Graphical Abstract. TME-EA extract was found to significantly inhibit melanin synthesis, exhibit anti-tyrosinase activity, and down-regulate some genes playing an important role in the melanin synthesis pathway. The amount of luteolin in TME-EA was analyzed using HPLC analysis, indicating that luteolin may be the main ingredient of TME fractions exerting anti-melanogenic effects. Additionally, we confirmed that the anti-melanogenic effect of TME-EA was mediated through the CREB/MAPK signaling pathway. Through further research that can address some limitations, it will be possible to extend the application area *T. maritima* extract as a cosmetic and functional whitening component.

## Figures and Tables

**Table 1:** RT-qPCR primer sequences for target genes.

Target Gene	Sequence (5′–3′)	
*Tyr*	Forward	AAG AAT GCT GCC CAC CAT GG
	Reverse	CAC GGT CAT CCA CCC CTT TG
*Trp1*	Forward	CAG TGC AGC GTC TTC CTG AG
	Reverse	TTC CCG TGG GAG CAC TGT AA
*Dct*	Forward	GAT GGC GTG CTG AAC AAG GA
	Reverse	ATA AGG GCC ACT CCA GGG TC
*Mitf*	Forward	ATC CCA TCC ACC GGT CTC TG
	Reverse	CCG TCC GTG AGA TCC AGA GT
*Mc1r*	Forward	TCA TCG TCC TCT GCC CTC AG
	Reverse	GCA GCA CCT CCT TGA GTG TC
*Gapdh*	Forward	TTG GCA TTG TGG AAG GGC TC
	Reverse	ACC AGT GGA TGC AGG GAT GA

**Table 2:** HPLC gradient profile for *T. maritima* extracts.

Time	A (0.1% formic acid in H_2_O) %	B (acetonitrile) %
0 min	10	90
20 min	90	10
30 min	90	10
35 min	10	90
45 min	10	90

## Data Availability

The datasets used and/or analyzed are available from the corresponding author upon reasonable request.

## Supplementary Materials

The following supporting information can be downloaded at: https://www.mdpi.com/article/10.3390/md22120532/s1, Figure S1: HPLC-PDA chromatogram of luteolin, TME, and TME-EA at 346 nm with UV spectra of the arrowed peak (right corner of the chromatogram).
