# Supplementary material for: Triglochin maritima Extracts Exert Anti-Melanogenic Properties via the CREB/MAPK Pathway in B16F10 Cells
Source: Mar Drugs. 2024 Nov 27;22(12):532. doi: 10.3390/md22120532 (PMC11677928; doi:10.3390/md22120532)

Supplementary Figure S1

HPLC-PDA chromatogram of luteolin, TME, and TME-EA at 346 nm with UV spectra of the arrowed peak (right corner of the chromatogram). Luteolin standard (0.1 mg/mL) (A); TME (1 mg/mL) (B); TME-EA (1 mg/mL) (C).

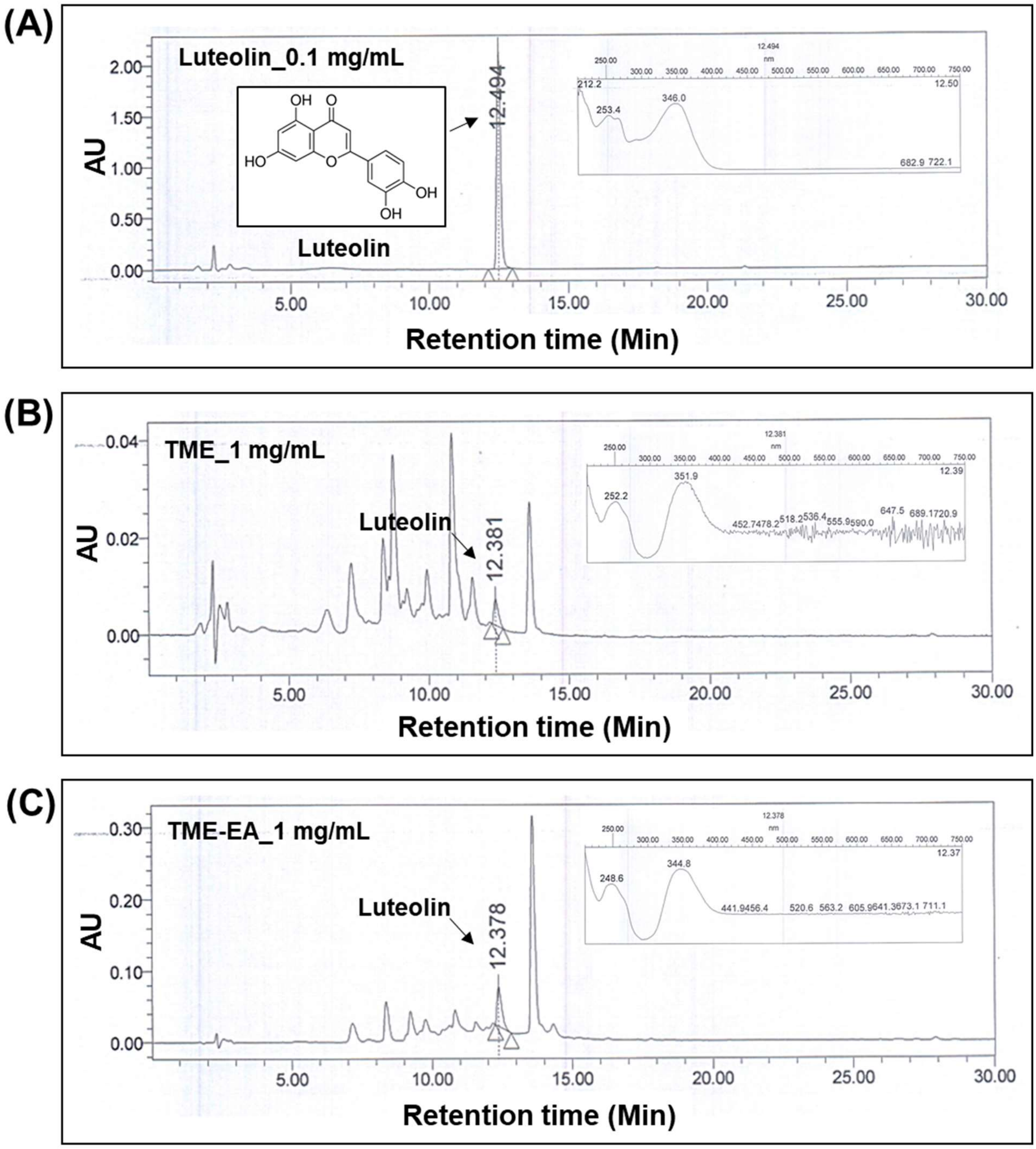

Supplement: Supplementary file 1 [file marinedrugs-22-00532-s001.zip › marinedrugs-3312223-supplementary.pdf]
